# Risk of Severe Acute Respiratory Syndrome Coronavirus 2 Acquisition Is Associated With Individual Exposure but Not Community-Level Transmission

**DOI:** 10.1093/infdis/jiac029

**Published:** 2022-02-02

**Authors:** DeAnna J Friedman-Klabanoff, Meagan C Fitzpatrick, Meagan E Deming, Vaidehi Agrawal, Sandra Sitar, Torin Schaafsma, Elizabeth Brown, Kathleen M Neuzil, Ruanne V Barnabas, Miriam K Laufer, Hydroxychloroquine COVID-19 Postexposure Prophylaxis Study Team, Peter Dull, Scott Miller, Thy Pham, Luisa Arroyave, Jonathan Berz, Pablo Buitron, Michael Camuso, Leticia Cardoso, Ricardo Cruz, Julien Dedier, Husam Dennaoui, Anna Goldman, Cheryl Greenstein, Lori Henault, Terrell Johnson, Sarah Kimball, Carlie Martinez, Erin Martinez, Crystal Ng, William Paarz, Qausarat Ogunneye, Lev Paasche‐Orlow, Margot Rogers, Kathleen Salerno, Michael Smithline, Carl Streed, Nisha Verma, Katherine Waite, Sybil Hosek, Christopher Balthazar, Ann Jarris, Leslie Anna Greene, Diana Buist, Sandra India Aldana, Marissa Alsaloum, Elle Anastasiou, Rodrigo Arce Cardozo, Beita Badiei, Kamilla Bakirova, Zulfiya Bakirova, Caroline Barnes, Sukhleen K Bedi, Dia B Beggs, Stefanie E Bendik, Meng Cao, Michelle Chang, Shirley Chen, Anna Cheng, Stella K Chong, Jane Coates, Sarah Conderino, Jacqueline Connery, Megan Connolly, Aira L Contreras, Matthew S Dreier, Emily Duan, Eveline Teresa Hidalgo, Maja Fadzan, Samantha N Fagan, Jamie Fried, Juan Gago, Nadia Gakou, Emily Gill, Akash Gujral, Xiaolei Hao, Christina Hughes, Robert T James, Sean Kim, Penina Krieger, Susan N Landon, Alice Li Juan Liang, Priscilla M Lopez, Lia Mamistvalova, Mark D Schwartz, Saydee McQuay, Wei (William) Miao, Sadia Mohaimin, Kaicy Gabriela, Naranjo, Krissy Nguyen‐Stone, Ashley Peltekci, Andrea Peña, Katherine Perdomo, Mary Lou Pompeii, Lindsey Quintana, Amanda J Reynolds, Robert A Pitts, Andrea Rodriguez, Prabhu Sasankan, Sneha R Sharma, Amy Shire, Stela Sota, Ben R Spoer, Bethany Springer, Jay Stadelman, Christina N Wysota, Jackie Yang, Myriam Yepez, Danielle Cram, Stephen Eustace, Kathleen Mandziuk, Michael Massaro, Colleen Mullenix, Toze Reichard, Tiara Towner, Mark Abbott, Elizabeth Asiago‐Reddy, Kristen Baxter, Kate Caiello, Timothy Endy, Ivayla Geneva, Peter Greco, Elizabeth Harausz, Michelle Klick, Patrick Mehlek, Christopher Miller, Melissa Reale, Kianna Ripich, Andrea Shaw, Keely Terrillion, Stephen Thomas, Mueenah Anibaba, Evan Atkinson, Michelle Blyth, Mary Beth Campbell, Lillianna Carsch, Ashita Ganguly, Sarina Gupta, Heather Larkin, Jake Hall, John Huntwork, Margaret Huntwork, Mariel McConville, April McDougal, Florice Numbi, Cedrick Ntambwe, Michelle Palomares, Demetrius Plaxico, Hamada Rady, Maria Ribando, Sydney Sauter, Daniel Triggs, Neha Upadhyay, Norine Schmidt, Shannon Watson, Crystal Zheng, Rubi Arias, Azra J Bhimani, Cherie Blair, Catie Cambou, Meilani Cayabyab, Rafael Corona, Danielle Escobedo, Daisuke Furukawa, Amanda Gonzalez, Heather Karpf, Ryan Kofron, Karla Largaespada, Nancy Lopez, Hannah Mansky, Rachel Martin‐Blais, Antonia Petreuse, Christina Shin, Tran T Tran, Ameila Weldon, Gabriela Were, Vaidehi Agrawal, Melissa Billington, Megan Birkhold, Colleen Boyce, Marianne Cloeren, Carlo Foppiano Palacios, DeAnna Friedman-Klabanoff, Alyson Kwon, Hanna LeBuhn, Esther Liu, Meredith Lu, Melissa McDiarmid, Melissa Myers, Faith Pa’ahana‐Brown, Marian Poley, Biraj Shrestha, Gentry Wilkerson, Nathan Alidina, Samuel Arnold, Marie Bauer, Jennifer Baugh, Cara Bayer, Medhavi Bole, Elizabeth Brandstetter, Alyssa Braun, Clare E Brown, Maxwell Brown, Michelle Bulterys, Jared Castor, Maianna Dematteis, Ivy Doan, Mark Drummond, Erika Feutz, Sean Galagan, Daphne Hamilton, Kirsten Hauge, Elsa Hay, Florian Hladik, Xuanlin Hou, Doug Houston, Madelaine Humphreys, Abir Hussein, Matthew Ikuma, Rodal Issema, Rachel Johnson, Mary Kirk, Jack Knauer, Steven R Kuntz, Savannah Lawton, Rebecca Letterer, Elina Lingappa, Jairam R Lingappa, Caroline H Liou, Katie Lund, Toni Maddox, Anya Mathur, Mari Metter, Lindsey McClellan, Deidra Montoya, Jessica C Moreno, Gregory Morlin, Urvashi Pandey, Gregory Pepper, Alicia Pettit, Thepthara Pholsena, Griffin Popp, Jeff Purcell, Justice Quame‐Amaglo, Reigran Sampoleo, Elliott Sanger, Matthew Seymour, Alexander Shercliffe, Shabir Somani, Jenell C Stewart, Selorm Tamakole, Jina M Taub, Zoe Thuesmunn, Amena Tungara, Ethan Valinetz, Dana L Varon, Vianey Vazquez, Bao‐Chau Vo, Valentine Wanga, Chloe D Waters, Yulun Wei, Meagan Welsh, Katie Wicklander, Brian R Wood, Grant Young, Zohdi Young, Lucy Zhao, Azaad Zimmermann, Deborah J Brown, Nathaniel Davenport, Omar Gambito

**Affiliations:** 1 Center for Vaccine Development and Global Health, University of Maryland School of Medicine, Baltimore, Maryland, USA; 2 Vaccine Research Center, National Institute of Allergy and Infectious Diseases, National Institutes of Health, Bethesda, Maryland, USA; 3 Department of Global Health, University of Washington, Seattle, Washington, USA; 4 Department of Biostatistics, University of Washington, Seattle, Washington, USA; 5 Vaccine and Infectious Disease Division, University of Washington, Seattle, Washington, USA; 6 Public Health Sciences Division, Fred Hutchinson Cancer Research Center, Seattle, Washington, USA; 7 Division of Allergy and Infectious Diseases, University of Washington, Seattle, Washington, USA; 8 Department of Epidemiology, University of Washington, Seattle, Washington, USA

**Keywords:** SARS-CoV-2, COVID-19, household transmission, healthcare worker transmission

## Abstract

**Background:**

Transmission rates after exposure to a severe acute respiratory syndrome coronavirus 2 (SARS-CoV-2)–positive individual within households and healthcare settings varies significantly between studies. Variability in the extent of exposure and community SARS-CoV-2 incidence may contribute to differences in observed rates.

**Methods:**

We examined risk factors for SARS-CoV-2 infection in a randomized controlled trial of hydroxychloroquine as postexposure prophylaxis. Study procedures included standardized questionnaires at enrollment and daily self-collection of midturbinate swabs for SARS-CoV-2 polymerase chain reaction testing. County-level incidence was modeled using federally sourced data. Relative risks and 95% confidence intervals were calculated using modified Poisson regression.

**Results:**

Eighty-six of 567 (15.2%) household/social contacts and 12 of 122 (9.8%) healthcare worker contacts acquired SARS-CoV-2 infection. Exposure to 2 suspected index cases (vs 1) significantly increased risk for both household/social contacts (relative risk [RR], 1.86) and healthcare workers (RR, 8.18). Increased contact time also increased risk for healthcare workers (3–12 hours: RR, 7.82, >12 hours: RR, 11.81, vs ≤2 hours), but not for household/social contacts. County incidence did not impact risk.

**Conclusions:**

In our study, increased exposure to SARS-CoV-2 within household or healthcare settings led to higher risk of infection, but elevated community incidence did not. This reinforces the importance of interventions to decrease transmission in close contact settings.

## Teaser

In an analysis of cases of SARS-CoV-2 transmission in a postexposure prophylaxis trial, increased exposure to SARS-CoV-2 within household or healthcare settings led to higher risk of infection, but higher community incidence did not.

As of 7 January 2022, more than 300 million cases and 5.5 million deaths have been attributed to severe acute respiratory syndrome coronavirus 2 (SARS-CoV-2) worldwide [1]. Viral shedding in the respiratory tract is high early in the infection, when symptoms are absent or mild [2, 3]. Due to this feature, contact between infected and susceptible people often occurs prior to diagnosis, complicating accurate identification of the transmission chain.

In studies of household and healthcare worker transmission, secondary cases of SARS-CoV-2 are typically attributed to the identified index case. However, in communities with high incidence, these secondary cases could alternatively have been community acquired. This misclassification would lead to inaccurate calculation of attack rates within households and healthcare settings. Systematic reviews of household transmission studies found that secondary attack rates vary widely from study to study, ranging from 4% to 55% [4–6]. Secondary attack rates in healthcare workers were lower (0–7%) but also variable [5]. The variability in secondary attack rates may be due to differences in the extent of exposure to index cases or may be a result of community acquisition of SARS-CoV-2 infection that was misclassified as household or healthcare-associated transmission.

Variability in these studies may also arise from differential risk factors in the study population. Among household contacts, known risk factors for SARS-CoV-2 acquisition include age, contact with a symptomatic compared to asymptomatic case, and more frequent contact with an index case [4–8]. However, the impact of risk factors such as sex and number of index case contacts on acquisition are uncertain [4, 6–8]. For healthcare contacts, most studies have focused on the role of personal protective equipment (PPE) in preventing infection, so other risk factors including age, sex, and ethnicity have been less well studied [9–11].

In a randomized clinical trial of the efficacy of hydroxychloroquine as postexposure prophylaxis for preventing SARS-CoV-2 infection, we observed an overall attack rate of 14% over 13 days among those who were not infected with SARS-CoV-2 at baseline [12]. Information gathered at enrollment and daily SARS-CoV-2 nasal swabs offered an opportunity to address gaps in knowledge about social, demographic, and health-related risk factors. We also used modeled community SARS-CoV-2 infection incidence to examine its effect on risk of infection. The overall aim was to identify risk factors for SARS-CoV-2 transmission in high-risk settings to inform public health strategies.

## METHODS

### Study Participants

Between March and August 2020, we recruited participants from across the United States to take part in a double-blind, household-randomized controlled trial of hydroxychloroquine as postexposure prophylaxis to prevent SARS-CoV-2 acquisition after close contact with an infected person (ClinicalTrials.gov identifier NCT04328961) [13]. The study occurred prior to the availability of SARS-CoV-2 vaccination. Close contact was defined as sharing a residence; prolonged (>15 minutes) exposure in a confined space; or, for healthcare workers, caring for a patient without appropriate PPE (mask, eye protection, gown, and gloves). We enrolled participants between 18 and 80 years of age whose contact occurred within the prior 96 hours and who could participate in the study visits via telehealth. Participants were excluded if they already displayed symptoms consistent with SARS-CoV-2, were hospitalized, or had contraindications to hydroxychloroquine. Before any study procedures were performed, all participants provided informed consent via telehealth and electronic signature. At enrollment, participants were asked standardized demographic, socioeconomic, and health behavior questions. Further description of the study and overall results have been published previously [12, 13].

### Outcome Measures

Within 2 days of study enrollment, participants received a box via courier containing study medication, sample collection supplies (nasal swabs and collection tubes with phosphate buffer solution transport medium), instructions, and packing materials for return. Participants were instructed to collect a midturbinate swab at the time of study box arrival and daily for the next 13 days. The packaged swabs were collected by a courier at several time points throughout the participation period and shipped back to the University of Washington for reverse-transcription polymerase chain reaction (RT-PCR) testing. A transmission episode was defined as a PCR-detectable SARS-CoV-2 infection in a participant who was SARS-CoV-2 negative at baseline.

The University of Washington Virology Laboratory conducted the RT-PCR testing using the primer sets N1 and N2 targeting the SARS-CoV-2 nucleocapsid [14]. Assays were performed with an ABI 7500 real-time PCR system (Applied Biosystems). A positive test was defined as having either or both N1 and N2 RNA detected with a cycle threshold value of ≤40. Further details regarding specimen collection and laboratory procedures have been described previously [13].

### Statistical Analysis

#### Local Incidence Data

County-level data on population size, daily number of COVID-19 cases and deaths, 7-day SARS-CoV-2 test positivity, and hospital inpatient bed use were sourced from the Health and Human Services data platform, HHS Protect [15]. To estimate the county-level incidence, we applied a formula that considered the daily number of confirmed cases, test positivity, number of days since 12 February 2020 (14 days before the first confirmed community transmission in the United States), and 3 constants derived from prior data about test positivity, serological surveys, hospitalization data, and number of deaths [16]. The constants adjust for limited test availability early in the pandemic.

### Regression

To be included, participants needed to have a negative baseline swab. Household/social contacts and healthcare workers were analyzed separately. Relative risks (RRs) and 95% confidence intervals (CIs) were calculated using modified Poisson regression for binary outcomes with robust standard errors (ie, generalized estimating equations), accounting for within-household clustering. The multivariate models include the primary exposure of interest (log_10_ daily county incidence rate) and all other treatment, demographic, socioeconomic, exposure, symptom status, and household characteristics of interest with a univariate *P* < .1, excluding characteristics with significant results for very small groups (n < 5 total or n < 1 infected contact per group). Daily study incidence rates were calculated using the number of participants with their first positive PCR result on that calendar day divided by the number of participants at risk on that day. The smoothed study incidence rate curve and 95% CI were calculated using a Poisson generalized additive model of the number of positives with an offset for the log number at risk. Daily county incidence rates are the mean of the modeled rates in the county of each participant at risk on that day (the same average rate for the participant’s 14-day follow-up period is used for each day a participant is at risk). The smoothed county incidence rate curve was calculated using local regression weighted by the number at risk. Statistical analyses were performed in R software, version 4.0.

## RESULTS

### Household/Social Contacts

A total of 567 household/social contacts were negative for SARS-CoV-2 at baseline. Participant characteristics are displayed in Table 1. The median age of the study population was 39 years (interquartile range [IQR], 25–51 years), 58% were female, and 77% lived with the participant. While 75% of our study population had only 1 known index case contact, 13% had 2 and 11% had >2. Eighty-six of the household/social contacts (15.2%) tested positive for SARS-CoV-2 by PCR during follow-up.

**Table 1.** Characteristics of Household/Social Contacts by Severe Acute Respiratory Syndrome Coronavirus 2 Acquisition Status

Characteristic	Total (N = 567)	SARS-CoV-2 Negative (n = 481)	SARS-CoV-2 Positive (n = 86)
Daily county incidence per 100 000 residents, median (IQR)	42.0 (22.3–87.5)	42.1 (22.3–83.4)	41.5 (22.1–97.3)
Randomization arm			
Ascorbic acid	277 (49)	236 (49)	41 (48)
Hydroxychloroquine	290 (51)	245 (51)	45 (52)
Age, y, median (IQR)	39 (25–51)	40 (25–51)	38 (26.25–51.75)
Age group, y			
18–24	129 (23)	110 (23)	19 (22)
25–34	113 (20)	97 (20)	16 (19)
35–44	96 (17)	80 (17)	16 (19)
45–54	127 (22)	110 (23)	17 (20)
55–64	73 (13)	58 (12)	15 (17)
65–80	29 (5)	26 (5)	3 (3)
Sex recorded at birth			
Female	327 (58)	281 (58)	46 (53)
Male	240 (42)	200 (42)	40 (47)
Race/ethnicity			
White (non-Hispanic)	287 (51)	256 (53)	31 (36)
Hispanic	143 (25)	116 (24)	27 (31)
Asian	58 (10)	43 (9)	15 (17)
Black or African American	46 (8)	36 (7)	10 (12)
Other	21 (4)	20 (4)	1 (1)
American Indian or Alaska Native	12 (2)	10 (2)	2 (2)
Education level			
Never graduated high school	25 (4)	19 (4)	6 (7)
High school graduate or GED	89 (16)	73 (15)	16 (19)
More than high school	453 (80)	389 (81)	64 (74)
Contact smokes	49 (9)	47 (10)	2 (2)
No. of suspected index cases			
1	428 (75)	377 (78)	51 (59)
2	75 (13)	57 (12)	18 (21)
>2	64 (11)	47 (10)	17 (20)
Symptomatic index case	409 (72)	340 (71)	69 (80)
Lived with index case for past 14 d	439 (77)	366 (76)	73 (85)
Hours of exposure to index case			
≤2	83/488 (17)	73/409 (18)	10/79 (13)
3–12	137/488 (28)	116/409 (28)	21/79 (27)
13–48	116/488 (24)	98/409 (24)	18/79 (23)
>48	152/488 (31)	122/409 (30)	30/79 (38)
Median (IQR)	20 (4–68)	16 (4–64)	36 (4.5–80)
Housing type			
House/condo/townhouse	410 (72)	350 (73)	60 (70)
Apartment	131 (23)	110 (23)	21 (24)
Dormitory/fraternity/sorority	21 (4)	17 (4)	4 (5)
Other	5 (1)	4 (1)	1 (1)
Total household members, median (IQR)	4 (2–5)	4 (3–5)	4 (2–5)
Household members per bathroom			
≤1	128/479 (27)	22 (26)	128/479 (27)
1.1–2	183/479 (38)	28 (33)	183/479 (38)
>2	168/479 (35)	36 (42)	168/479 (35)
Median (IQR)	2 (1–3)	2 (1–3)	2 (1.06–3)
Household members per bedroom			
≤1	273/565 (48)	238/479 (50)	35 (41)
1.1–2	240/565 (42)	196/479 (41)	44 (51)
>2	52/565 (9)	45/479 (9)	7 (8)
Median (IQR)	1.25 (1–1.67)	1.2 (1–1.67)	1.33 (1–2)
Any children in household	243/566 (43)	199/480 (41)	44 (51)

Data are presented as No. (%) unless otherwise indicated. Denominator is provided if any missing data.

Abbreviations: GED, General Educational Development; IQR, interquartile range; SARS-CoV-2, severe acute respiratory syndrome coronavirus 2.

### Risk Factors for SARS-CoV-2 Infection in Household/Social Contacts

Table 2 displays the univariate relative risk of SARS-CoV-2 infection by selected risk factors. As was reported previously, randomization arm (hydroxychloroquine vs ascorbic acid) did not affect risk of SARS-CoV-2 infection [12]. Symptomatic infection in the index case also did not increase participant risk of SARS-CoV-2 infection despite similar mean hours of exposure to the index case in the prior 4 days: 34.3 hours for those with a symptomatic index case compared with 37.6 hours for those with an asymptomatic index case (*t* test *P* = .45). Participant identification as Hispanic, Asian, or Black/African American was associated with higher risk of SARS-CoV-2 infection relative to participant identification as non-Hispanic White (RRs, 1.75, 2.39, and 2.01, respectively). Smoking was associated with a lower risk of SARS-CoV-2 infection with an RR of 0.25 (95% CI, .06–.99). Having multiple suspected index cases was associated with a higher risk of SARS-CoV-2 infection: RRs, 2.01 (95% CI, 1.23–3.29) for 2 suspected index cases and 2.23 (95% CI, 1.36–3.64) for >2 suspected index cases.

**Table 2.** Relative Risk of Severe Acute Respiratory Syndrome Coronavirus 2 Infection in a Household/Social Contact by Select Factors^a^

Characteristic	no./No. (%)	Univariate Analysis		Multivariate Analysis	
		RR (95% CI)	*P* Value	RR (95% CI)	*P* Value
Daily county incidence per resident (log_10_)		1.13 (.73–1.76)	.584	1.07 (.68–1.71)	.763
Randomization arm					
Ascorbic acid	41/277 (14.8)	ref			
Hydroxychloroquine	45/290 (15.5)	1.05 (.70–1.57)	.818	Not included	
Age (in decades)		1.01 (.89–1.15)	.844	Not included	
Sex recorded at birth					
Female	46/327 (14.1)	ref			
Male	40/240 (16.7)	1.18 (.80–1.76)	.401	Not included	
Race/ethnicity					
White (non-Hispanic)	31/287 (10.8)	ref		ref	
Hispanic	27/143 (18.9)	1.75 (1.07–2.87)	.027^∗^	1.54 (.91–2.60)	.104
Asian	15/58 (25.9)	2.39 (1.38–4.14)	.002^∗∗^	2.02 (1.16–3.50)	.012^∗^
Black or African American	10/46 (21.7)	2.01 (1.06–3.83)	.033^∗^	1.71 (.89–3.31)	.109
Other	1/21 (4.8)	0.44 (.06–3.11)	.411	0.39 (.05–2.96)	.363
American Indian or Alaska Native	2/12 (16.7)	1.54 (.41–5.84)	.523	0.88 (.24–3.28)	.849
Highest level of education					
Never graduated high school	6/25 (24.0)	ref			
High school graduate or GED	16/89 (18.0)	0.75 (.34–1.66)	.476		
More than high school	64/453 (14.1)	0.59 (.29–1.18)	.133	Not included	
Contact smokes					
No	84/518 (16.2)	ref		ref	
Yes	2/49 (4.1)	0.25 (.06–.99)	.049^∗^	0.28 (.07–1.09)	.067
No. of suspected index cases					
1	51/428 (11.9)	ref		ref	
2	18/75 (24.0)	2.01 (1.23–3.29)	.005^∗∗^	1.86 (1.13–3.07)	.015^∗^
>2	17/64 (26.6)	2.23 (1.36–3.64)	.001^∗∗^	1.90 (1.18–3.08)	.009^∗∗^
Symptomatic index case					
No	17/158 (10.8)	ref		ref	
Yes	69/409 (16.9)	1.57 (.95–2.58)	.076	1.42 (.85–2.37)	.183
Lived with index case for past 14 d					
No	13/128 (10.2)	ref		ref	
Yes	73/439 (16.6)	1.64 (.95–2.83)	.077	1.34 (.77–2.33)	.304
Hours of exposure to index case					
≤2	10/83 (12.0)	ref			
3–12	21/137 (15.3)	1.27 (.67–2.41)	.461		
13–48	18/116 (15.5)	1.29 (.64–2.60)	.481		
>48	30/152 (19.7)	1.64 (.85–3.15)	.139	Not included	
Housing type					
House/condo/townhouse	60/410 (14.6)	ref			
Apartment	21/131 (16.0)	1.10 (.69–1.74)	.701		
Dormitory/fraternity/sorority	4/21 (19.0)	1.30 (.52–3.25)	.572		
Other	1/5 (20.0)	1.37 (.23–8.05)	.730	Not included	
No. of household occupants		1.01 (.99–1.02)	.571	Not included	
Occupants per bedroom					
≤1	35/273 (12.8)	ref		ref	
1.1–2	44/240 (18.3)	1.43 (.94–2.18)	.097	1.27 (.82–1.98)	.282
>2	7/52 (13.5)	1.05 (.48–2.29)	.903	0.80 (.34–1.89)	.617
Occupants per bathroom					
≤1	22/150 (14.7)	ref			
1.1–2	28/211 (13.3)	0.90 (.54–1.51)	.702		
>2	36/204 (17.6)	1.20 (.73–1.98)	.466	Not included	
Any children in household					
No	42/323 (13.0)	ref			
Yes	44/243 (18.1)	1.39 (.93–2.08)	.107	Not included	

Abbreviations: CI, confidence interval; GED, General Educational Development; ref, reference group; RR, relative risk.

Only variables with a *P* < .01 and with at least 1 infected contact per group were included in the multivariate model.

∗*P* < .05, ∗∗*P* < .01.

The multivariate model included 565 of the 567 participants from 455 of 456 households, excluding 2 participants with missing data. Variables included in the multivariate analysis were county incidence rate, participant race/ethnicity, participant smoking, number of suspected index cases, symptomatic index case (prior to the time of enrollment), living with index case in the 14 days prior to enrollment, and number of occupants per bedroom in the household (Table 2). In the multivariate model, only Asian race and exposure to >1 index case remained significant. Participant identification as Asian was associated with an adjusted RR (aRR) of SARS-CoV-2 infection of 2.02 (95% CI, 1.16–3.50) relative to non-Hispanic White. Having multiple suspected index cases was associated with an aRR of SARS-CoV-2 infection of 1.86 (95% CI, 1.13–3.07) for 2 suspected index cases and aRR of 1.90 (95% CI, 1.18–3.08) for >2 suspected index cases.

### Study Incidence and Local Incidence Rates

**Figure 1.**
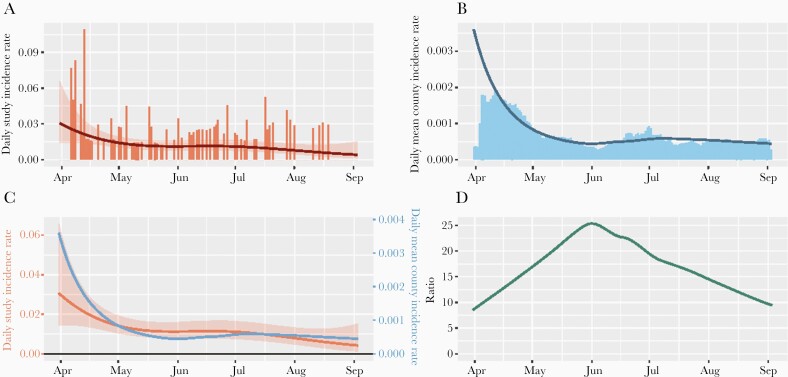
Study and county daily incidence rates for household/social contacts. *A*, Study incidence rates per participant, by calendar day, in counties where participants resided with smoothed curve and 95% confidence interval (Poisson generalized additive model). *B*, Mean modeled county incidence rates per resident, by calendar day, with smoothed loess curve. *C*, Smoothed curves for study (red) and county (blue) incidence rates with y-axes normalized to the mean. *D*, Ratio of smoothed study to county incidence rates. While county incidence rates mirrored the overall high incidence seen in the spring of 2020 and lower incidence seen in the summer of 2020 in most areas of the United States, study incidence rates decreased over time.

The mean modeled daily county incidence rate was 42.0 (IQR, 22.2–87.5) daily new infections per 100 000 residents during follow-up in the counties where participants were located. Figure 1 displays study incidence rate and mean county incidence rate by calendar day and comparisons between the 2. While the county incidence rate was higher in spring 2020 and lower in summer 2020, the study incidence rate decreased steadily and gradually over time. No significant difference was detected between the daily mean county incidence rates for those who acquired SARS-CoV-2 infection during follow-up and those who did not (Table 2).

### Healthcare Worker Contacts

We enrolled 122 healthcare worker contacts who were negative for SARS-CoV-2 at baseline. The median age was 37 years (IQR, 31–47 years), and 70% were female. Table 3 displays participant characteristics. Most of this population had multiple exposures: 34% had a single known index case contact, 11% had 2, and 55% had >2. Twelve (9.8%) tested positive for SARS-CoV-2 by PCR from a nasal swab during follow-up.

**Table 3.** Characteristics of Healthcare Worker Contacts by Severe Acute Respiratory Syndrome Coronavirus 2 Acquisition Status

Characteristic	Total (N = 122)	SARS-CoV-2 Negative (n = 110)	SARS-CoV-2 Positive (n = 12)
Daily county incidence per 100 000 residents, median (IQR)	29.8 (16.1–81.7)	30.5 (15.4–81.7)	23.2 (18.5–61.0)
Randomization arm			
Ascorbic acid	59 (48)	55 (50)	4 (33)
Hydroxychloroquine	63 (52)	55 (50)	8 (67)
Age, y, median (IQR)	37 (31–47)	37 (30.25–46.75)	35 (33–50.5)
Age group, y			
18–24	10 (8)	9 (8)	1 (8)
25–34	39 (32)	34 (31)	5 (42)
35–44	33 (27)	32 (29)	1 (8)
45–54	27 (22)	24 (22)	3 (25)
55–64	13 (11)	11 (10)	2 (17)
65–80	0 (0)	0 (0)	0 (0)
Sex recorded at birth			
Female	85 (70)	76 (69)	9 (75)
Male	37 (30)	34 (31)	3 (25)
Race/ethnicity			
White (non-Hispanic)	82 (67)	75 (68)	7 (58)
Hispanic	12 (10)	10 (9)	2 (17)
Asian	15 (12)	12 (11)	3 (25)
Black or African American	8 (7)	8 (7)	0 (0)
Other	5 (4)	5 (5)	0 (0)
American Indian or Alaska Native	0 (0)	0 (0)	0 (0)
Education level			
Never graduated high school	0 (0)	0 (0)	0 (0)
High school graduate or GED	4 (3)	2 (2)	2 (17)
More than high school	118 (97)	108 (98)	10 (83)
Contact smokes	10 (8)	8 (7)	2 (17)
No. of suspected indexes			
1	41 (34)	40 (36)	1 (8)
2	14 (11)	10 (9)	4 (33)
>2	67 (55)	60 (55)	7 (58)
Hours of exposure to index case			
≤2	57 (47)	56 (51)	1 (8)
3–12	34 (28)	29 (26)	5 (42)
>12	31 (25)	25 (23)	6 (50)
Median (range)	3.5 (1–14.25)	2 (1–12)	14 (6–24)
Housing type			
House/condo/townhouse	94 (77)	84 (76)	10 (83)
Apartment	22 (18)	21 (19)	1 (8)
Dormitory/fraternity/sorority	1 (1)	0 (0)	1 (8)
Other	5 (4)	5 (5)	0 (0)
Bedrooms in household			
0–1	16/121 (13)	15/109 (14)	1 (8)
2	26/121 (21)	25/109 (23)	1 (8)
3	45/121 (37)	40/109 (37)	5 (42)
>3	34/121 (28)	29/109 (27)	5 (42)
Bathrooms in household			
1	36/121 (30)	34/109 (31)	2 (17)
2	43/121 (36)	39/109 (36)	4 (33)
>2	42/121 (35)	36/109 (33)	6 (50)

Data are presented as No. (%) unless otherwise indicated. Denominator is provided if any missing data.

Abbreviations: GED, General Educational Development; IQR, interquartile range; SARS-CoV-2, severe acute respiratory syndrome coronavirus 2.

### Risk Factors for SARS-CoV-2 Infection in Healthcare Worker Contacts

The univariate RRs of SARS-CoV-2 infection in healthcare worker contacts by selected risk factors are displayed in Table 4. None of the participants who identified their race as Black/African American or other acquired SARS-CoV-2 infection, although the numbers of participants in these groups were small. Participants who had more than a high school level of education were less likely to acquire SARS-CoV-2 (RR, 0.17 [95% CI, .04–.65]). Exposure to 2 suspected index cases increased risk of SARS-CoV-2 infection (RR, 11.71 [95% CI, 1.41–97.02]) compared with 1 index case, but having >2 suspected index cases did not. However, only 1 infection occurred in the reference group, which may have affected findings. Increasing exposure time also increased risk of SARS-CoV-2 acquisition; relative to <3 hours of exposure, the RR of 3–12 hours was 8.38 (95% CI, 1.02–68.81), and the RR of >12 hours of exposure was 11.03 (95% CI, 1.34–90.55).

**Table 4.** Relative Risk of Severe Acute Respiratory Syndrome Coronavirus 2 Infection in Healthcare Worker Contacts by Select Factors^a^

Factor	no./No. (%)	Univariate Model		Multivariate Model	
		RR (95% CI)	*P* Value	RR (95% CI)	*P* Value
Daily county incidence per resident (log_10_)		0.89 (.33–2.39)	.812	0.84 (.28–2.49)	.749
Randomization arm					
Ascorbic acid	4/59 (6.8)	ref			
Hydroxychloroquine	8/63 (12.7)	1.87 (.51–6.83)	.342	Not included	
Age (in decades)		1.19 (.67–2.11)	.562	Not included	
Sex recorded at birth					
Female	9/85 (10.6)	ref			
Male	3/37 (8.1)	0.77 (.24–2.44)	.652	Not included	
Race/ethnicity					
White (non-Hispanic)	7/82 (8.5)	ref			
Hispanic	2/12 (16.7)	1.95 (.46–8.30)	.365		
Asian	3/15 (20.0)	2.34 (.61–8.96)	.214		
Black or African American	0/8 (0)	0.00 (.00–.00)	<.0001^∗∗∗^		
Other	0/5 (0)	0.00 (.00–.00)	<.0001^∗∗∗^	Not included	
Highest level of education					
High school graduate or GED	2/4 (50.0)	ref			
More than high school	10/118 (8.5)	0.17 (.04–.65)	.010^∗^	Not included	
Contact smokes					
No	10/112 (8.9)	ref			
Yes	2/10 (20.0)	2.24 (.56–9.01)	.256	Not included	
No. of suspected index cases					
1	1/41 (2.4)	ref		ref	
2	4/14 (28.6)	11.71 (1.41–97.02)	.023^∗^	8.18 (1.26–53.32)	.028^∗^
>2	7/67 (10.4)	4.28 (.55–33.57)	.166	1.92 (.34–11.03)	.463
Hours of exposure to index case					
≤2	1/57 (1.8)	ref		ref	
3–12	5/34 (14.7)	8.38 (1.02–68.81)	.048^∗^	7.82 (1.09–56.21)	.041^∗^
>12	6/31 (19.4)	11.03 (1.34–90.55)	.025^∗^	11.81 (2.00–69.70)	.006^∗∗^
Housing type					
House/condo/townhouse	10/94 (10.6)	ref			
Apartment	1/22 (4.5)	0.43 (.06–3.20)	.408		
Dormitory/fraternity/sorority	1/1 (100.0)	9.40 (5.03–17.57)	<.0001^∗∗∗^		
Other	0/5 (0)	0.00 (.00–.00)	<.0001^∗∗∗^	Not included	
Bedrooms in household					
0–1	1/16 (6.2)	ref			
2	1/26 (3.8)	0.62 (.04–9.17)	.725		
3	5/45 (11.1)	1.78 (.22–14.10)	.586		
>3	5/34 (14.7)	2.35 (.29–19.18)	.424	Not included	
Bathrooms in household					
1	2/36 (5.6)	ref			
2	4/43 (9.3)	1.67 (.32–8.63)	.538		
>2	6/42 (14.3)	2.57 (.53–12.37)	.239	Not included	

Abbreviations: CI, confidence interval; GED, General Educational Development; RR, relative risk.

Only variables with a *P* < .01, a size of at least 5 per group, and at least 1 infected contact per group were included in the multivariate model.

∗*P* < .05, ∗∗*P* < .01, ∗∗∗*P* < .001.

Variables included in the multivariate model were county incidence rate, number of suspected index cases, and hours of exposure to an index case (Table 4). Having 2 suspected index cases and hours of exposure to the index case remained significant in the multivariate model. The adjusted RR of SARS-CoV-2 acquisition after contact with 2 suspected index cases compared to 1 index case was 8.18 (95% CI, 1.26–53.32). Compared to ≤2 hours of contact with the index case, having 3–12 hours of contact was associated with an aRR of 7.82 (95% CI, 1.09–56.21) and having >12 hours of contact was associated with an aRR of 11.81 (95% CI, 2.00–69.70).

### Healthcare Worker Incidence and Local Incidence

**Figure 2.**
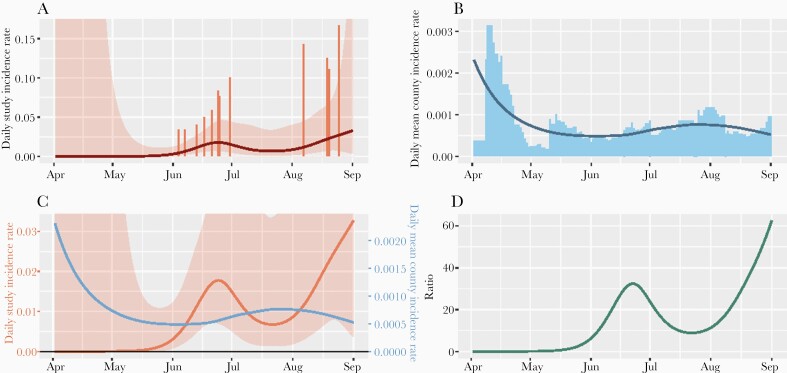
Study and county daily incidence rates for healthcare worker contacts. *A*, Study incidence rates per participant, by calendar day, with smoothed curve and 95% confidence interval (Poisson generalized additive model). *B*, Mean modeled county incidence rate per resident, by calendar day, with smoothed loess curve. *C*, Smoothed curves for study (red) and county (blue) incidence rates with y-axes normalized to the mean. *D*, Ratio of smoothed curves for study and county incidence rates.

The mean modeled county daily incidence rate was 29.8 (IQR, 16.1–81.7) per 100 000 residents during follow-up of healthcare worker contacts. Figure 2 displays study and county incidence rates similar to Figure 1, but for healthcare workers. While most of the cases of SARS-CoV-2 infection in the study occurred in June and August and increased over time, county incidence rates were relatively low during these times compared to earlier in the study. The surrounding daily mean county incidence rates were not different for healthcare workers who acquired SARS-CoV-2 infection and those who did not (Table 4).

## DISCUSSION

In this national, multicenter prospective study, we found that increased exposure, but not local infection incidence, contributed to the risk of SARS-CoV-2 acquisition for household contacts. This epidemiological data supports the conclusion of many studies identifying household exposure as a primary source of risk, even when community incidence is relatively high. To our knowledge, our study provides one of the most detailed quantifications of factors that contribute to risk of household transmission with the most comprehensive postexposure testing. We identified multiple suspected index cases as the key independent risk factor for household transmission. The lack of association between the reported time spent with the index case and successful transmission could represent incomplete isolation of index cases in the household; indicate that small amounts of intense exposure, such as caregiving, pose a large risk; or that exposure prior to the test result, which might not have been captured in our survey, led to much of the transmission. All these potential possibilities suggest the need for more aggressive interventions to limit exposure when there is an index case identified in a household. The report of time spent could also be subject to recall bias, but the categories for analysis were wide, which would mitigate the effect of some inaccuracy.

The lack of association between local incidence and study incidence confirms that an infection within the household posed far greater short-term risk than infection in the surrounding community and has implications for our understanding of how mitigation policies affect household spread. Prior studies have shown that containment measures such as lockdown, distance learning, telework, and closure of nonessential businesses decrease the proportion of SARS-CoV-2 infections acquired in the community and increase the proportion of infections acquired in the household, even though total number of infections may be lower [17, 18]. However, the measures in many previous studies were much more extreme than those introduced in the United States.

As expected, the time spent in contact with an index case was associated with risk of transmission for healthcare workers. Our finding about the importance of multiple exposures is novel. Although having >2 exposures was not statistically significantly associated with transmission, we suspect this can be attributed to the small sample size in this cohort. Little prior information exists regarding the effect of multiple high-risk exposures on risk of SARS-CoV-2 infection for healthcare workers, as this variable is not often included in studies [10]. In the absence of optimal PPE, our results support extensive efforts to limit the time providers spend with SARS-CoV-2–infected patients.

Interestingly, contact with a symptomatic index case did not increase risk of SARS-CoV-2 infection compared to an asymptomatic index case, contrasting with prior literature [4–7]. Due to limited use of routine SARS-CoV-2 screening at the time of the study, identification of asymptomatic infections was rare, and >70% of our participants had been exposed to a symptomatic index case. At enrollment, participants were asked about hours spent with the index within the last 96 hours; this number did not differ between those with a symptomatic index case and those with an asymptomatic index case, but the time from onset of symptoms to diagnosis in the index case and time from diagnosis to enrollment varied. We were also not able to account for differences in quarantining behaviors (eg, whether the index and contact used a shared kitchen and/or bathroom, used masks in the home) or ventilation of the household, factors which could have affected outcomes. Also, participants were asked about index case symptoms at the time of enrollment. It is possible that some of the index cases became symptomatic after the participant was enrolled and thus were misclassified.

Participant identification as Asian also increased risk of SARS-CoV-2 infection in household/social contacts, which has not consistently been seen in prior studies [4–6]. These participants were enrolled throughout the course of the study, but 41% of Asian participants were from the Seattle area. Therefore, some of the cases could have been epidemiologically linked. However, only 2 of the 15 infected lived in the same ZIP (postal) code. We were not able to incorporate specific comorbidities into the model due to small numbers, so this could also have affected risk. Also, we note that Asian heritage includes a broad range of ethnic and socioeconomic backgrounds, so the information we gathered may not be precise enough to fully understand the implications of this finding. The inconsistent effect of race and ethnicity on risk across this study and others previously published suggests that race/ethnicity may not be the independent risk factor but represents unmeasured cofounding that we have not captured.

Some of the subgroups for healthcare workers contained very small numbers, including some categories of race/ethnicity, level of education, and housing type, making it difficult to draw conclusions about their effect on risk. The method of recruitment and completion of study procedures also likely selected for participants with a higher socioeconomic status, as is reflected in the high proportion of participants with more than a high school education. This may limit the generalizability of the findings to a more diverse population. Also, we analyzed county-level data for SARS-CoV-2 incidence rates due to the data available. However, finer-resolution data at the ZIP code or census tract level may have been more representative of an individual’s actual risk.

In this national, multicenter study in the era prior to vaccine deployment and the emergence of highly transmissible variants, we found that regardless of changing community-level transmission or mitigation behaviors, limiting exposure to a known SARS-CoV-2–infected household member or patient remains a critical approach to interrupting transmission. Our novel study design incorporated community incidence into the model and found no significant association between this metric and the probability of transmission within households in the setting of a known close contact. However, widespread community mitigation measures present in most of the United States during the period of the study may have affected these findings, as contact with others outside of the household was limited. It is unclear at this time if the effect of community transmission on household transmission would change in the absence of mitigation measures, which currently vary across the country. As cases rise again due to variant emergence coinciding with increased in-person workplace and school interactions, more support for aggressive mitigation measures aimed at household and healthcare settings is critical to limit spread of SARS-CoV-2.

## Notes

***Acknowledgments.*** We thank the study participants, our collaborators for this project, and the Hydroxychloroquine COVID-19 Postexposure Prophylaxis Study Team for their hard work and dedication to this study.

***Financial support.*** This work was supported by the Bill & Melinda Gates Foundation through the COVID-19 Therapeutics Accelerator (INV-016204) and the University of Washington King K. Holmes Endowed Professorship in STDs and AIDS. Additional support was received from the National Institutes of Health (award numbers K23AI155838 to D. J. F.-K., K01 AI141576 to M. C. F., and K24AI14996 to M. K. L.). Hydroxychloroquine for the study was donated by Sandoz.

***Potential conflicts of interest.*** All authors: No reported conflicts of interest.

## APPENDIX

***Hydroxychloroquine COVID-19 Preexposure Prophylaxis Study Team members (listed in alphabetical order by institution).*** Bill & Melinda Gates Foundation: Peter Dull, Scott Miller, Thy Pham; Boston University School of Medicine: Luisa Arroyave, Jonathan Berz, Pablo Buitron, Michael Camuso, Leticia Cardoso, Ricardo Cruz, Julien Dedier, Husam Dennaoui, Anna Goldman, Cheryl Greenstein, Lori Henault, Terrell Johnson, Sarah Kimball, Carlie Martinez, Erin Martinez, Crystal Ng, William Paarz, Qausarat Ogunneye, Lev Paasche‐Orlow, Margot Rogers, Kathleen Salerno, Michael Smithline, Carl Streed, Nisha Verma, Katherine Waite; Cook County Health: Sybil Hosek, Christopher Balthazar; Discovery Health: Ann Jarris; Everett Clinic: Leslie Anna Greene; Kaiser Permanente: Diana Buist; New York University Grossman School of Medicine: Sandra India Aldana, Marissa Alsaloum, Elle Anastasiou, Rodrigo Arce Cardozo, Beita Badiei, Kamilla Bakirova, Zulfiya Bakirova, Caroline Barnes, Sukhleen K. Bedi, Dia B. Beggs, Stefanie E. Bendik, Meng Cao, Michelle Chang, Shirley Chen, Anna Cheng, Stella K. Chong, Jane Coates, Sarah Conderino, Jacqueline Connery, Megan Connolly, Aira L. Contreras, Matthew S. Dreier, Emily Duan, Eveline Teresa Hidalgo, Maja Fadzan, Samantha N. Fagan, Jamie Fried, Juan Gago, Nadia Gakou, Emily Gill, Akash Gujral, Xiaolei Hao, Christina Hughes, Robert T. James, Sean Kim, Penina Krieger, Susan N. Landon, Alice Li Juan Liang, Priscilla M. Lopez, Lia Mamistvalova, Mark D. Schwartz, Saydee McQuay, Wei (William) Miao, Sadia Mohaimin, Kaicy Gabriela Naranjo, Krissy Nguyen‐Stone, Ashley Peltekci, Andrea Peña, Katherine Perdomo, Mary Lou Pompeii, Lindsey Quintana, Amanda J. Reynolds, Robert A. Pitts, Andrea Rodriguez, Prabhu Sasankan, Sneha R. Sharma, Amy Shire, Stela Sota, Ben R. Spoer, Bethany Springer, Jay Stadelman, Christina N. Wysota, Jackie Yang, Myriam Yepez; PRA Health Sciences: Danielle Cram, Stephen Eustace, Kathleen Mandziuk, Michael Massaro, Colleen Mullenix, Toze Reichard, Tiara Towner; State University of New York Upstate Medical University: Mark Abbott, Elizabeth Asiago‐Reddy, Kristen Baxter, Kate Caiello, Timothy Endy, Ivayla Geneva, Peter Greco, Elizabeth Harausz, Michelle Klick, Patrick Mehlek, Christopher Miller, Melissa Reale, Kianna Ripich, Andrea Shaw, Keely Terrillion, Stephen Thomas; Tulane University: Mueenah Anibaba, Evan Atkinson, Michelle Blyth, Mary Beth Campbell, Lillianna Carsch, Ashita Ganguly, Sarina Gupta, Heather Larkin, Jake Hall, John Huntwork, Margaret Huntwork, Mariel McConville, April McDougal, Florice Numbi, Cedrick Ntambwe, Michelle Palomares, Demetrius Plaxico, Hamada Rady, Maria Ribando, Sydney Sauter, Daniel Triggs, Neha Upadhyay, Norine Schmidt, Shannon Watson, Crystal Zheng; University of California, Los Angeles: Rubi Arias, Azra J. Bhimani, Cherie Blair, Catie Cambou, Meilani Cayabyab, Rafael Corona, Danielle Escobedo, Daisuke Furukawa, Amanda Gonzalez, Heather Karpf, Ryan Kofron, Karla Largaespada, Nancy Lopez, Hannah Mansky, Rachel Martin‐Blais, Antonia Petreuse, Christina Shin, Tran T. Tran, Ameila Weldon, Gabriela Were); University of Maryland School of Medicine: Vaidehi Agrawal, Melissa Billington, Megan Birkhold, Colleen Boyce, Marianne Cloeren, Carlo Foppiano Palacios, DeAnna Friedman‐Klabanoff, Alyson Kwon, Hanna LeBuhn, Esther Liu, Meredith Lu, Melissa McDiarmid, Melissa Myers, Faith Pa’ahana‐Brown, Marian Poley, Biraj Shrestha, Gentry Wilkerson; University of Washington: Nathan Alidina, Samuel Arnold, Marie Bauer, Jennifer Baugh, Cara Bayer, Medhavi Bole, Elizabeth Brandstetter, Alyssa Braun, Clare E. Brown, Maxwell Brown, Michelle Bulterys, Jared Castor, Maianna Dematteis, Ivy Doan, Mark Drummond, Erika Feutz, Sean Galagan, Daphne Hamilton, Kirsten Hauge, Elsa Hay, Florian Hladik, Xuanlin Hou, Doug Houston, Madelaine Humphreys, Abir Hussein, Matthew Ikuma, Rodal Issema, Rachel Johnson, Mary Kirk, Jack Knauer, Steven R. Kuntz, Savannah Lawton, Rebecca Letterer, Elina Lingappa, Jairam R. Lingappa, Caroline H. Liou, Katie Lund, Toni Maddox, Anya Mathur, Mari Metter, Lindsey McClellan, Deidra Montoya, Jessica C. Moreno, Gregory Morlin, Urvashi Pandey, Gregory Pepper, Alicia Pettit, Thepthara Pholsena, Griffin Popp, Jeff Purcell, Justice Quame‐Amaglo, Reigran Sampoleo, Elliott Sanger, Matthew Seymour, Alexander Shercliffe, Shabir Somani, Jenell C. Stewart, Selorm Tamakole, Jina M. Taub, Zoe Thuesmunn, Amena Tungara, Ethan Valinetz, Dana L. Varon, Vianey Vazquez, Bao‐Chau Vo, Valentine Wanga, Chloe D. Waters, Yulun Wei, Meagan Welsh, Katie Wicklander, Brian R. Wood, Grant Young, Zohdi Young, Lucy Zhao, Azaad Zimmermann; Virginia Mason Memorial Hospital: Deborah J. Brown, Nathaniel Davenport, Omar Gambito.
